# Antibiotic Resistance in Vibrio cholerae: Mechanistic Insights from IncC Plasmid-Mediated Dissemination of a Novel Family of Genomic Islands Inserted at *trmE*

**DOI:** 10.1128/mSphere.00748-20

**Published:** 2020-08-26

**Authors:** Nicolas Rivard, Rita R. Colwell, Vincent Burrus

**Affiliations:** a Département de Biologie, Université de Sherbrooke, Sherbrooke, Québec, Canada; b Maryland Pathogen Research Institute, University of Maryland, College Park, Maryland, USA; c John Hopkins Bloomberg School of Public Health, John Hopkins University, Baltimore, Maryland, USA; d Center for Bioinformatics and Computational Biology, University of Maryland, College Park, Maryland, USA; JMI Laboratories

**Keywords:** antibiotic resistance, IncC plasmids, mobilization, relaxase, T4CP, T4SS, *Vibrio cholerae*, conjugation, genomic islands, horizontal gene transfer, *oriT*, phage resistance

## Abstract

Cholera remains a formidable disease, and reports of multidrug-resistant strains of the causative agent Vibrio cholerae have become common during the last 3 decades. The pervasiveness of resistance determinants has largely been ascribed to mobile genetic elements, including SXT/R391 integrative conjugative elements, IncC plasmids, and genomic islands (GIs). Conjugative transfer of IncC plasmids is activated by the master activator AcaCD whose regulatory network extends to chromosomally integrated GIs. MGI*Vch*Hai6 is a multidrug resistance GI integrated at the 3′ end of *trmE* (*mnmE* or *thdF*) in chromosome 1 of non-O1/non-O139 V. cholerae clinical isolates from the 2010 Haitian cholera outbreak. In the presence of an IncC plasmid expressing AcaCD, MGI*Vch*Hai6 excises from the chromosome and transfers at high frequency. Herein, the mechanism of mobilization of MGI*Vch*Hai6 GIs by IncC plasmids was dissected. Our results show that AcaCD drives expression of GI-borne genes, including *xis* and *mobI_M_*, involved in excision and mobilization. A 49-bp fragment upstream of *mobI_M_* was found to serve as the minimal origin of transfer (*oriT*) of MGI*Vch*Hai6. The direction of transfer initiated at *oriT* was determined using IncC plasmid-driven mobilization of chromosomal markers via MGI*Vch*Hai6. In addition, IncC plasmid-encoded factors, including the relaxase TraI, were found to be required for GI transfer. Finally, *in silico* exploration of *Gammaproteobacteria* genomes identified 47 novel related and potentially AcaCD-responsive GIs in 13 different genera. Despite sharing conserved features, these GIs integrate at *trmE*, *yicC*, or *dusA* and carry a diverse cargo of genes involved in phage resistance.

**IMPORTANCE** The increasing association of the etiological agent of cholera, Vibrio cholerae serogroup O1 and O139, with multiple antibiotic resistance threatens to deprive health practitioners of this effective tool. Drug resistance in cholera results mainly from acquisition of mobile genetic elements. Genomic islands conferring multidrug resistance and mobilizable by IncC conjugative plasmids were reported to circulate in non-O1/non-O139 V. cholerae clinical strains isolated from the 2010 Haitian cholera outbreak. As these genomic islands can be transmitted to pandemic V. cholerae serogroups, their mechanism of transmission needed to be investigated. Our research revealed plasmid- and genomic island-encoded factors required for the resistance island excision, mobilization, and integration, as well as regulation of these functions. The discovery of related genomic islands carrying diverse phage resistance genes but lacking antibiotic resistance-conferring genes in a wide range of marine dwelling bacteria suggests that these elements are ancient and recently acquired drug resistance genes.

## Abstract (precis)

The increasing association of the etiological agent of cholera, Vibrio cholerae serogroup O1 and O139, with multiple antibiotic resistance threatens to deprive health practitioners of this effective tool. Drug resistance in cholera results mainly from acquisition of mobile genetic elements. Genomic islands conferring multidrug resistance and mobilizable by IncC conjugative plasmids were reported to circulate in non-O1/non-O139 V. cholerae clinical strains isolated from the 2010 Haitian cholera outbreak. As these genomic islands can be transmitted to pandemic V. cholerae serogroups, their mechanism of transmission needed to be investigated. Our research revealed plasmid- and genomic island-encoded factors required for the resistance island excision, mobilization, and integration, as well as regulation of these functions. The discovery of related genomic islands carrying diverse phage resistance genes but lacking antibiotic resistance-conferring genes in a wide range of marine dwelling bacteria suggests that these elements are ancient and recently acquired drug resistance genes.

## INTRODUCTION

Cholera is an acute diarrheal disease that leads to severe dehydration and often death in the absence of adequate treatment (1). The seventh cholera pandemic, which began in 1961, is caused by toxigenic strains of the *Gammaproteobacteria* Vibrio cholerae serogroup O1 biotype El Tor, or more sporadically its O139 variant (1). Since the late 1980s, antibiotic-resistant V. cholerae strains have emerged and spread globally (2). Development of drug resistance in seventh pandemic V. cholerae has been ascribed to mutation but it mostly involves acquisition of mobile genetic elements, including genomic island GI-15, integrative conjugative elements of the SXT/R391 family, and conjugative plasmids of incompatibility group C (IncC) (3–5).

IncC plasmids are large (>120-kbp) broad-host-range conjugative plasmids frequently associated with multidrug resistance in several species of globally distributed pathogenic enterobacteria, and in seventh pandemic V. cholerae strains from Africa, China, and Haiti (4, 6–8). Conjugative transfer of IncC plasmids is controlled by the FlhCD-like heteromeric transcriptional activator AcaCD that they encode (9). AcaCD activates 17 promoters conserved in IncC plasmids, including those driving expression of transfer genes and operons encoding type IV secretion system (T4SS) and conjugative pilus, relaxase TraI, putative type IV coupling protein (T4CP) TraD, and unknown function protein TraJ (9). TraI belongs to the MOB_H1_ family of relaxases, and together with the product of *mobI_C_*, is essential for initiation of conjugative transfer at the origin of transfer (*oriT*) (10, 11). MobI_C_ is responsible for recognition of the *oriT* locus of IncC plasmids that is located immediately upstream of *mobI_C_* (12). Unlike other transfer genes, *mobI_C_* seems to be expressed in an AcaCD-independent manner (9).

**FIG 1:**
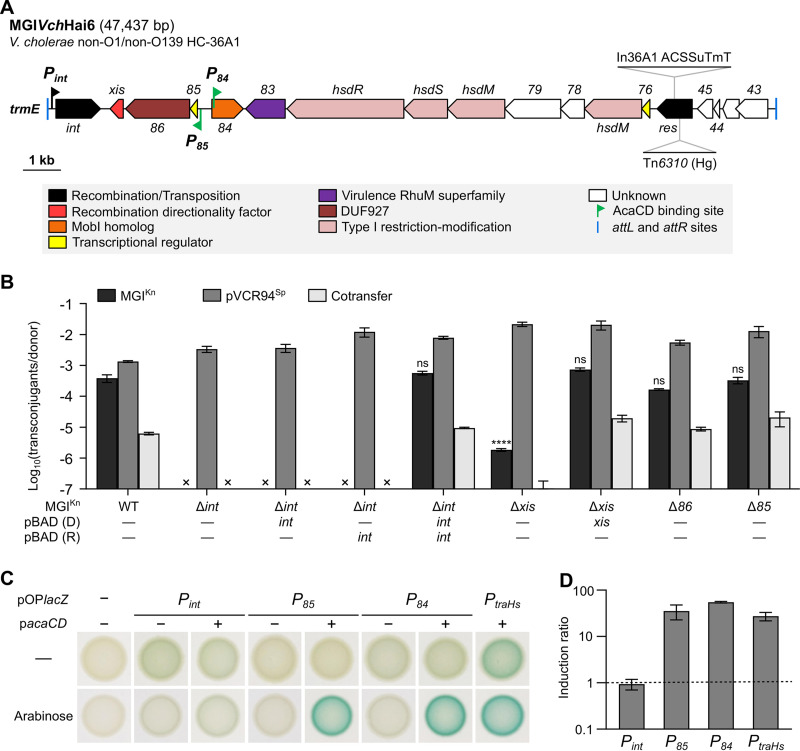
Role and regulation of *int* and *85-86*-*xis* in the mobilization of MGI*Vch*Hai6. (A) Schematic genetic map of MGI*Vch*Hai6 drawn to scale. The left and right junctions (*attL* and *attR*) within the host chromosome are indicated by blue ticks at the extremities. ORFs with similar function are color coded as indicated in the figure. Green flags indicate the position and orientation of predicted AcaCD binding sites (13). The black flag indicates the position and orientation of the *P_int_* promoter. The insertion sites of In36A1 integron and Tn*6310* transposon are shown. The gene numbers correspond to the last digits of the respective locus tags in GenBank accession no. AXDR01000001. ACSSuTmT, resistance to ampicillin, chloramphenicol, spectinomycin/streptomycin, sulfamethoxazole, trimethoprim, and tetracycline; Hg, mercury resistance. (B) Mobilization assays of MGI^Kn^ or its Δ*int*, Δ*xis*, Δ*86*, or Δ*85* mutants were carried out using E. coli GG56 (Nx) bearing pVCR94^Sp^ as the donor strain, and CAG18439 (Tc) as the recipient strain. Complementation assays were performed in the donor (D) or recipient (R) strain by expressing the missing gene from *P_BAD_* on pBAD-*int* or pBAD-*xis*. An “×” indicates that the transfer frequency was below the detection limit (<10^−7^). Bars represent the means ± standard errors of the means (error bars) from three independent experiments. Statistical analyses were carried out on the logarithm of the values using a one-way analysis of variance (ANOVA), followed by Dunnett’s multiple-comparison test with the wild-type (WT) MGI^Kn^ as the control. Statistical significance is indicated as follows: ****, *P* < 0.0001; ns, not significant. (C) β-Galactosidase activities of *P_int_*, *P_85_*, and *P_84_* transcriptionally fused to *lacZ*. Colonies were grown on LB agar with or without arabinose to induce *acaCD* expression from p*acaCD*. (D) Induction levels of *P_int_*, *P_85_*, and *P_84_* in response to AcaCD. β-Galactosidase assays were carried out using the strains of panel C. Ratios between the enzymatic activities in Miller units for the arabinose-induced versus noninduced strains containing p*acaCD* are shown. The AcaCD-regulated promoter *P_traHs_* of SGI1 served as a positive control, and cells devoid of pOP*lacZ* served as a negative control. The bars represent the means ± standard errors of the means (error bars) from two independent experiments.

Furthermore, AcaCD also triggers excision of at least three types of genomic islands (GIs) shown to be mobilizable in *trans* by IncC plasmids. The first type, integrated at the 3′ end of the gene of unknown function *yicC*, is exemplified by the 16.5-kbp MGI*Vmi*1 of Vibrio mimicus (9). The two other types of GIs are inserted into the 3′ end of *trmE* (also known as *mnmE* or *thdF*), a gene encoding the 5-carboxymethylaminomethyluridine-tRNA synthase GTPase subunit (13, 14). One type of GI is illustrated by the 42.4-kbp *Salmonella* genomic island 1 (SGI1) that confers resistance to ampicillin, chloramphenicol, streptomycin/spectinomycin, sulfamethoxazole, and tetracycline (ACSSuT) in Salmonella enterica (14, 15). The other is illustrated by the 47.4-kbp MGI*Vch*Hai6 of V. cholerae HC-36A1 that confers not only the ACSSuT phenotype but also trimethoprim and possibly mercury resistance (Fig. 1A) (13). MGI*Vch*Hai6 was found in non-O1/non-O139 V. cholerae strains isolated from patients exhibiting symptoms of cholera at the onset of the 2010 cholera outbreak in Haiti. Despite integrating into the same site as SGI1, MGI*Vch*Hai6 encodes a distantly related integrase Int (67% identity) and recombination directionality factor (RDF) Xis (37% identity) (13). MGI*Vch*Hai6 also lacks most of the genes and sequences that enable the mobilization of SGI1 by IncC plasmids (13, 16, 17). On the basis of these structural differences, the mechanisms of mobilization of MGI*Vch*Hai6 and SGI1 by IncC plasmids are expected to differ considerably. In MGI*Vch*Hai6, *xis* is the last gene of a putative operon-like structure preceded by a putative AcaCD-controlled promoter (Fig. 1A). A second AcaCD binding site is located inside an open reading frame (ORF) that encodes a distant homolog of MobI_C_ (27% identity over two fragments of 109 and 53 amino acid residues) (13). Besides AcaCD, IncC plasmid- and GI-encoded factors involved in MGI*Vch*Hai6 mobilization have not been characterized.

In this report, we established a model of mobilization of MGI*Vch*Hai6 by helper IncC plasmids and compared this model to SGI1 mobilization. Deletion mutants of the helper plasmid and MGI were used in mating assays to characterize the contribution of each element in MGI*Vch*Hai6 mobilization. The presence of AcaCD binding sites upstream of *xis* and inside the putative gene encoding a MobI_C_ homolog suggests the presence of AcaCD-responsive promoters that were verified using *lacZ* reporter fusions. By analogy with IncC plasmids, we hypothesized that the MobI_C_ homolog encoded by MGI*Vch*Hai6 recognizes a cognate *oriT* locus located upstream of its gene. The ability of IncC plasmids to mobilize chromosomal DNA through MGI*Vch*Hai6 was also tested and provided the directionality of transfer initiated at *oriT*. Finally, phylogenetic analyses based on the mobilization factor revealed the existence of a large class of potential IncC-mobilized GIs that are integrated at three different chromosomal sites in mostly marine dwelling species of *Gammaproteobacteria*. Our results show that MGI*Vch*Hai6-like GIs share a mechanism of mobilization by helper IncC plasmids that differs from the one used by SGI1-like GIs.

## RESULTS

### *int* and *xis* are essential for excision and mobilization of MGI*Vch*Hai6.

MGI*Vch*Hai6 carries a large cargo of antibiotic and mercury resistance genes. To make it more amenable for this study, we constructed a kanamycin (Kn)-resistant mutant of MGI*Vch*Hai6 that lacks In36A1, Tn*6310*, and *res* (Fig. 1A). The resulting 19.5-kb mutant, named hereafter MGI^Kn^, was mobilized by the helper IncC plasmid pVCR94^Sp^ at the same frequency as MGI*Vch*Hai6 ([5.2 ± 0.3] × 10^−4^ versus [5.7 ± 1.2] × 10^−4^, *P* = 0.622, Student’s *t* test).

To establish whether *int* is required for mobilization of MGI*Vch*Hai6, we carried out mobilization assays using pVCR94^Sp^ and a Δ*int* mutant of MGI^Kn^. Deletion of *int* abolished MGI^Kn^ mobilization (Fig. 1B). Complementation assays done by expressing *int* from the arabinose-inducible promoter *P_BAD_* on pBAD-*int* restored mobilization to the wild-type level only when pBAD-*int* was present in both the donor and recipient strains. Therefore, *int* is likely required for excision of MGI^Kn^ in donor cells and for its integration into the chromosome of recipient cells.

The predicted (RDF) gene *xis* encodes a 114-amino-acid (aa)-residue protein containing a predicted helix-turn-helix (HTH_17, Pfam accession no. PF12728) domain (position 39 to 89). To assess the role of *xis*, we mobilized the Δ*xis* mutant of MGI^Kn^ using pVCR94^Sp^. Transfer of this mutant was reduced ∼200-fold compared to the wild type (Fig. 1B). Complementation using pBAD-*xis* in donor cells was sufficient to restore the mobilization of MGI^Kn^ Δ*xis* to the wild-type level, thereby confirming that Xis is required only in donor cells, likely to facilitate Int-mediated excision of the GI.

*xis* is located downstream of two open reading frames (ORFs), *85* and *86* (the gene numbers correspond to the last digits of the respective locus tags in GenBank accession no. AXDR01000001), of unknown function. The predicted translation products of *85* is a 65-aa-residue protein that, like *xis*, contains an HTH_17 domain (position 11 to 62). *86* encodes a predicted 558-aa-residue protein containing a domain of unknown function (DUF927, Pfam accession no. PF06048) in its N-terminal half. Mobilization assays using Δ*85* and Δ*86* mutants of MGI^Kn^ and pVCR94^Sp^ revealed that neither *85* nor *86* is involved in the excision, mobilization, or integration of MGI^Kn^ under laboratory conditions, as the frequency of mobilization remained unaffected by deletions (Fig. 1B).

To validate the involvement of *int* and *xis* in the excision step, we carried out PCR excision assays to detect the *attP* site on the plasmid-like form of excised MGI^Kn^, using E. coli GG56 (nalidixic acid) bearing MGI^Kn^ or MGI^Kn^ Δ*int*. Spontaneous excision was undetectable, as shown by the absence of *attP* PCR product (see Fig. S1 in the supplemental material). Overexpression of *int* from *P_int_* did not trigger excision, whereas overexpression of *xis* resulted in excision of MGI^Kn^, but not of MGI^Kn^ Δ*int*. Thus, excision of MGI*Vch*Hai6 is induced by Xis and requires Int, in line with their proposed roles of RDF and integrase, respectively.

10.1128/mSphere.00748-20.1 FIG S1 Ethidium bromide-stained 1.5% agarose gel of PCR products for detection of MGI^Kn^ excision. Excision assays were carried out in E. coli GG56 bearing MGI^Kn^ or its Δ*int* mutant and, where indicated, arabinose-inducible vector pBAD-*int* or pBAD-*xis*. Download FIG S1, PDF file, 0.08 MB. Copyright © 2020 Rivard et al. 2020 Rivard et al. This content is distributed under the terms of the Creative Commons Attribution 4.0 International license.

### AcaCD-dependent activation of mobility genes of MGI*Vch*Hai6.

AcaCD binding sites were previously detected upstream of *85* and inside *84* (13). To test whether AcaCD activates promoter sequences upstream of *int* and *85* or inside *84*, *P_int_*, *P_85_*, and *P_84_* were introduced upstream of a single-copy, chromosomal promoterless *lacZ* gene cassette that is transcriptionally isolated by the terminator sequences *rgnB* and *tl3* (9). The AcaCD-responsive promoter sequence *P_traHs_* of SGI1 (17) was used as a positive control. The β-galactosidase activity of each promoter was monitored in the presence and absence of ectopically expressed *acaCD*.

*P_int_* yielded a weak yet constitutive β-galactosidase activity regardless of the presence of AcaCD (Fig. 1C), confirming that the promoter that drives expression of the integrase gene is not controlled by AcaCD. In contrast, *P_85_*, which likely drives expression of the RDF gene *xis*, did not appear to produce detectable β-galactosidase activity in the absence of AcaCD (Fig. 1C). When *acaCD* was expressed, *P_85_* activity increased 35-fold (Fig. 1D). Finally, *P_84_* exhibited a weak, constitutive expression similar to *P_int_* and weaker than *P_traHs_* in the absence of AcaCD (Fig. 1C). Like *P_traHs_*, *P_84_* was strongly induced (55-fold increase) upon expression of *acaCD* (Fig. 1D).

These results confirm that the two promoter sequences *P_85_* and *P_84_* containing the predicted AcaCD binding sites are activated by AcaCD. In contrast, *P_int_* drives low-level constitutive expression of the integrase gene.

### MobI_M_ is required for MGI*Vch*Hai6 mobilization.

**FIG 2:**
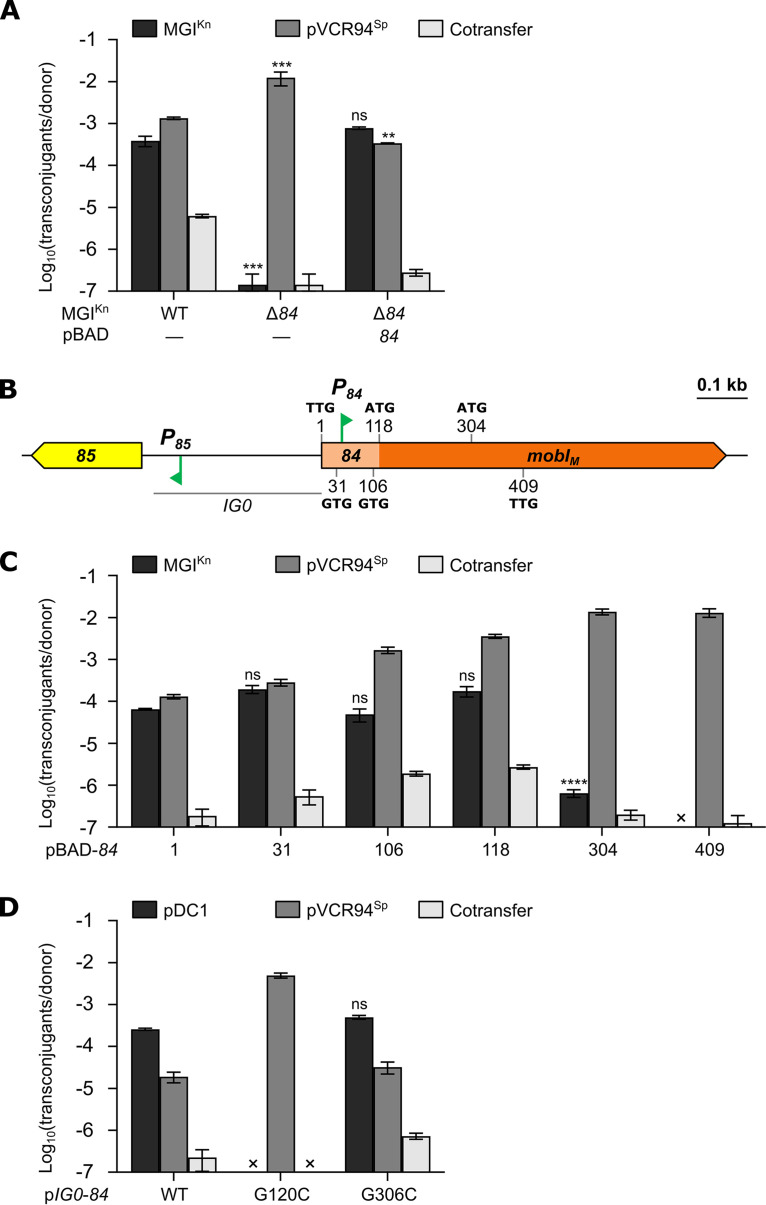
Role of *84* in MGI*Vch*Hai6 mobilization. (A) Mobilization of MGI^Kn^ or its Δ*84* mutant by pVCR94^Sp^. (B) Schematic representation of the *85-84* region of MGI*Vch*Hai6. Genes are color coded as indicated in Fig. 1A. The position and sequence of alternative start codons within *84* are indicated. (C) Complementation assays of the Δ*84* mutation by alternative ORFs within *84*. When indicated, donor strains contained the complementation plasmid pBAD-*84* or one of its derivatives (Table 1). (D) Confirmation of ATG_118_ as the genuine start codon of *mobI_M_*. Conjugation assays were performed using E. coli GG56 (Nx) containing the specified elements as donor strains and either CAG18439 (Tc) (A and C) or VB112 (Rf) (D) as the recipient strain. The bars represent the means ± standard errors of the means from three independent experiments. Statistical analyses were carried out on the logarithm of the values using a one-way ANOVA followed by Dunnett’s multiple-comparison test with the WT MGI^Kn^ (A), pBAD-*84* (C), or p*IG0*-*84* (D) as the control. Statistical significance is indicated as follows: ****, *P* < 0.0001; ***, *P* < 0.001; **, *P* < 0.01; ns, not significant.

The 795-bp open reading frame *84* (locus VCHC36A1_0084 of V. cholerae HC-36A1) of MGI*Vch*Hai6 encodes a distant homolog of MobI_C_ (27% identity). Since MobI_C_ is a key factor for IncC plasmid transfer, we wondered whether *84* could play an important role in mobilization of MGI*Vch*Hai6. To test the hypothesis, we constructed MGI^Kn^ Δ*84* and carried out mobilization assays with pVCR94^Sp^. MGI^Kn^ transfer was strongly impaired by this deletion and reduced ∼2,700-fold (Fig. 2A). In this context, we observed a slight (12-fold), yet statistically significant, increase of pVCR94^Sp^ transfer. Complementation of the Δ*84* mutation from pBAD-*84* restored MGI^Kn^ mobilization to the wild-type level, while reducing transfer of pVCR94^Sp^ ∼40-fold, suggesting that *84* is essential for MGI*Vch*Hai6 mobilization. However, the position of the AcaCD-responsive *P_84_* promoter inside *84* suggested that the protein effective for mobilization is smaller than predicted. To test this hypothesis, we constructed complementation plasmids containing fragments of *84* starting at the following alternative start codons: +31, GTG; +106, GTG; +118, ATG; +304, ATG; and +409, TTG (Fig. 2B). All plasmids but pBAD-*84-*304 and pBAD-*84*-409 restored mobilization of MGI^Kn^ Δ*84* by pVCR94^Sp^ to the wild-type level (Fig. 2C). Therefore, the ORF located downstream of *P_84_* and starting at ATG_118_ in *84* is the likely gene that allows MGI*Vch*Hai6 mobilization. This gene, hereafter referred to as *mobI_M_*, produces a putative 225-aa-residue protein.

### Localization and characterization of the origin of transfer (*oriT*).

In IncC plasmids, *oriT* is located directly upstream of *mobI_C_* (10, 12). By analogy, we predict that *oriT* of MGI*Vch*Hai6 is located at the corresponding position, i.e., upstream of *mobI_M_*. To confirm this hypothesis, we first cloned the intergenic region located between *85* and *84* (*IG0*) as well as *84* into the low-copy-number nonmobilizable plasmid pDC1. The resulting plasmid, p*IG0*-*84*, was mobilized by pVCR94^Sp^ at a frequency comparable to that of MGI^Kn^ (Fig. 2D), thereby confirming the cloned fragment sufficient to support mobilization by the IncC plasmid even in the absence of MGI*Vch*Hai6. Site-directed mutagenesis G120C (ATG to ATC) and G306C (ATG to ATC) in *84* confirmed that ATG_118_ is the start codon of *mobI_M_* since p*IG0*-*84* carrying the mutation G120C was not mobilizable, whereas mutation G306C had no effect on transfer (Fig. 2D).

**FIG 3:**
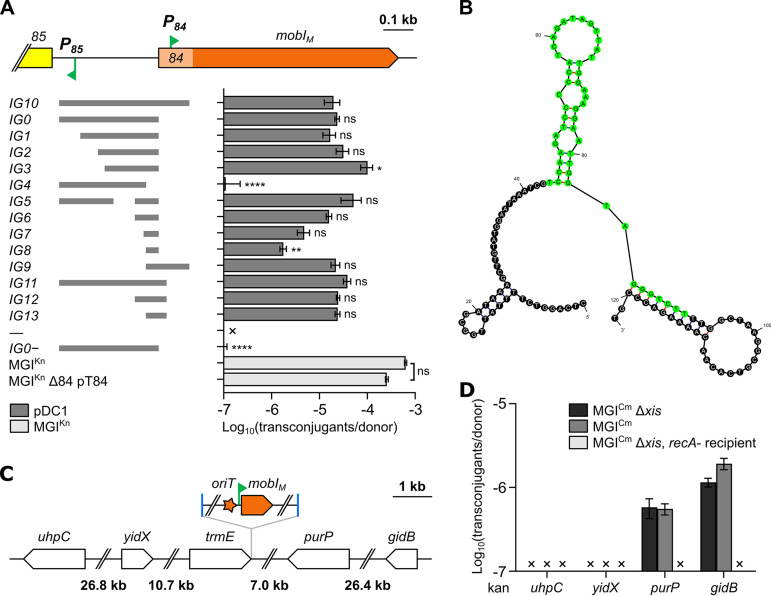
Localization of the *oriT* locus of MGI*Vch*Hai6. (A) On the left, fragments of the *85*-*mobI* region that were cloned into the nonmobilizable vector pDC1 are represented by gray bars. The resulting transfer frequencies for the corresponding fragments are presented on the right side. Mobilization assays of pDC1 derivatives were performed using E. coli GG56 (Nx) bearing pVCR94^Sp^ and pT84 as the donor and VB112 (Rf) as the recipient. “—” indicates an empty pDC1. “*IG0−*” indicates that the transfer of p*IG0* was assessed in the absence of pT84. Statistical analyses were carried out on the logarithm of the values using a one-way ANOVA followed by Dunnett’s multiple-comparison test with p*IG10* or MGI^Kn^ as the control. Statistical significance is indicated as follows: ****, *P* < 0.0001; **, *P* < 0.01; *, *P* < 0.05; ns, not significant. (B) Predicted folding of *IG12*, with *IG8* highlighted in green. Folding of the upper strand (panel A) was predicted using the Mfold web server (74). (C) Schematic map of the chromosomal region surrounding *trmE* in E. coli K-12. The position and orientation of MGI^Cm^ are indicated. (D) MGI^Cm^-mediated mobilization of chromosomal markers from E. coli JW3642, JW3692, JW3718, or JW5858 (Kn) bearing pVCR94^Sp^ and MGI^Cm^ or its Δ*xis* mutant to CAG18439 or its Δ*recA* mutant (Tc). In panels A and D, the bars represent the means ± standard errors of the means from three independent experiments. “×” indicates that the transfer frequency was below the detection limit (<10^−7^).

Since we were able to use pBAD-*84*-118 to complement and mobilize the Δ*84* mutant of MGI^Kn^ that lacks the 117-bp segment containing the AcaCD-responsive promoter *P_84_*, the possibility that *oriT* could be located in this sequence or in *mobI_M_* was ruled out. Therefore, we cloned *IG0* and the region extending upstream of *mobI_M_* (*IG10*) into pDC1 and carried out mobilization assays using pVCR94^Sp^ to provide the conjugative machinery. Donor cells also carried pT84 to provide MobI_M_. The empty vector and the same vector bearing *IG0* in the absence of pT84 were both unable to transfer (Fig. 3A). In contrast, when pT84 was present in donor cells, the vector bearing *IG10* or *IG0* transferred at frequencies that were comparable to that of MGI^Kn^ Δ*84* complemented with pT84. This result indicated that *oriT* is located within *IG0*.

Insert reduction was then performed to find the minimal sequence of *IG0* allowing mobilization by pVCR94^Sp^. The smallest insert capable of acting as a suitable *oriT*, *IG8*, was 49 bp long and located immediately upstream of *84* (Fig. 3A). Although functional, *IG8* provided only 1/10th of the mobilization activity of *IG0* or larger inserts such as *IG6* or *IG13* that provided additional upstream or downstream sequences. The addition of either 43 bp upstream (*IG6*) or 30 bp downstream (*IG13*) led to transfer frequencies equivalent to that obtained with *IG10*, and addition of both fragments (*IG12*) did not enhance transfer further. Predicted folding of *IG12* revealed three potential stem-loop structures within *IG8* and on either side, highlighting the presence of repeated sequences potentially involved in relaxosome assembly (Fig. 3B).

### Directionality of transfer initiated at *oriT*.

To determine the direction of conjugative transfer initiated at *oriT* of MGI*Vch*Hai6, chromosomal markers located upstream and downstream of *trmE*, the integration site of MGI*Vch*Hai6, were tested for mobilization. Accordingly, MGI^Cm^ or the excision-defective mutant MGI^Cm^ Δ*xis* were introduced together with pVCR94^Sp^ into Escherichia coli BW25113 derivatives carrying a kanamycin resistance (Kn^r^) marker integrated at *uhpC*, *yidX*, *purP*, or *gidB* (Keio knockout collection) (18). These genes are located between 7 and 39.5 kb on either side of *trmE* (Fig. 3C). Mobilization assays failed to produce any transconjugants when the Kn^r^ marker was inserted upstream of *trmE*. In contrast, transfer was easily detectable for Kn^r^ insertions at *purP* and *gidB* that are located downstream of *trmE*, regardless of the ability of MGI^Cm^ to excise from the chromosome (Fig. 3D). This result demonstrates that transfer of MGI*Vch*Hai6 initiated at *oriT* progresses downstream of *mobI_M_* and that the last genes translocated into the recipient cells are *xis*-*86*-*85*.

### Involvement of IncC DNA processing genes in MGI*Vch*Hai6 mobilization.

**FIG 4:**
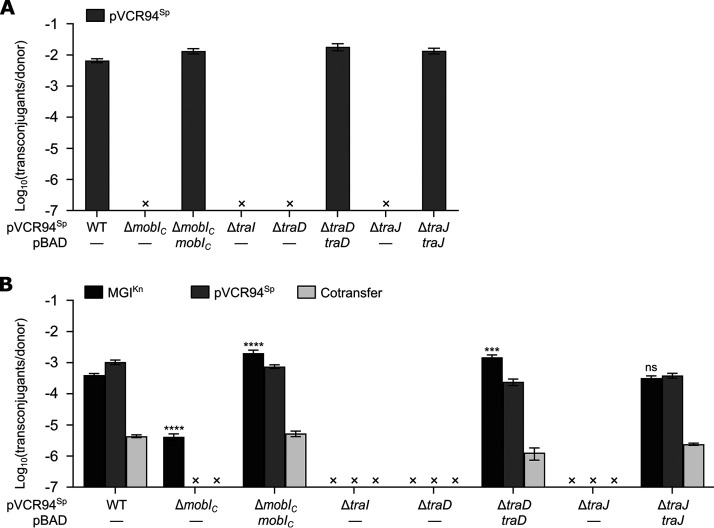
Mobilization of MGI*Vch*Hai6 relies on the T4CP and relaxase encoded by IncC plasmids. (A) Impact of *mobI_C_*, *traI*, *traD*, and *traJ* deletions on pVCR94^Sp^ transfer. (B) Mobilization of MGI^Kn^ by the *mobI_C_*, *traI*, *traD*, and *traJ* deletion mutants of pVCR94^Sp^. Conjugation assays were performed with E. coli GG56 (Nx) containing the specified elements as donor strains and CAG18439 (Tc) as the recipient strain. The bars represent the means ± standard errors of the means from three independent experiments. “×” indicates that the transfer frequency was below the detection limit (<10^−7^). Statistical analyses were carried out on the logarithm of the values using a one-way ANOVA followed by Dunnett’s multiple-comparison test with WT MGI^Kn^ as the control. Statistical significance is indicated as follows: ****, *P* < 0.0001; ***, *P* < 0.001; ns, not significant.

To test whether DNA processing genes of IncC plasmids are involved in the mobilization of MGI*Vch*Hai6, we constructed nonpolar deletion mutants of *mobI_C_*, *traI*, *traD*, and *traJ* in pVCR94^Sp^ and carried out conjugation assays. Each individual deletion abolished transfer of pVCR94^Sp^. Except for *traI*, all deletions could be complemented by ectopic expression of the corresponding gene (Fig. 4A). Complementation of *traI* could not be tested as attempts to clone an intact copy of this gene failed, suggesting that expression under *P_BAD_*, even in the absence of arabinose, is toxic.

As observed for pVCR94^Sp^, deletion of *traI*, *traD*, and *traJ* abolished MGI^Kn^ mobilization. Complementation of each mutation restored mobilization to the wild-type level (Fig. 4B). Therefore, these genes appear to be essential for processing and transfer of excised MGI^Kn^ DNA. Interestingly, although MGI*Vch*Hai6 encodes its own MobI_M_ factor, deletion of *mobI_C_* resulted in ∼100-fold reduction of MGI^Kn^ mobilization (Fig. 4B), whereas complementation with pBAD-*mobI*_C_ restored mobilization to the wild-type level.

Likewise, we confirmed that MGI*Vch*Hai6 relies exclusively on the T4SS encoded by IncC plasmids as deletion of *traH_C_*, *traG_C_*, or *traN_C_* abolished mobilization of MGI^Kn^ (Fig. S2). Moreover, mobilization of MGI^Kn^ by pVCR94^Sp^ into a recipient strain carrying pVCR94^Cm^ was abolished (Fig. S2), thus confirming that MGI*Vch*Hai6 does not evade IncC entry exclusion.

10.1128/mSphere.00748-20.2 FIG S2 Mobilization of MGI^Kn^ by pVCR94^Sp^ T4SS mutants. “pVCR94^Cm^ (R)” indicates that the element was present in the recipient strain. Donor and recipient strains are the same as in Fig. 4. Statistical analyses were carried out on the logarithm of the values using a one-way ANOVA followed by Dunnett’s multiple comparison test with WT MGI^Kn^ as the control. Statistical significance is indicated as follows: **, *P* < 0.01; ns, not significant. Download FIG S2, PDF file, 0.05 MB. Copyright © 2020 Rivard et al. 2020 Rivard et al. This content is distributed under the terms of the Creative Commons Attribution 4.0 International license.

### MGI*Vch*Hai6 is the prototype of a large and diverse subfamily of GIs mobilizable by IncC plasmids.

**FIG 5:**
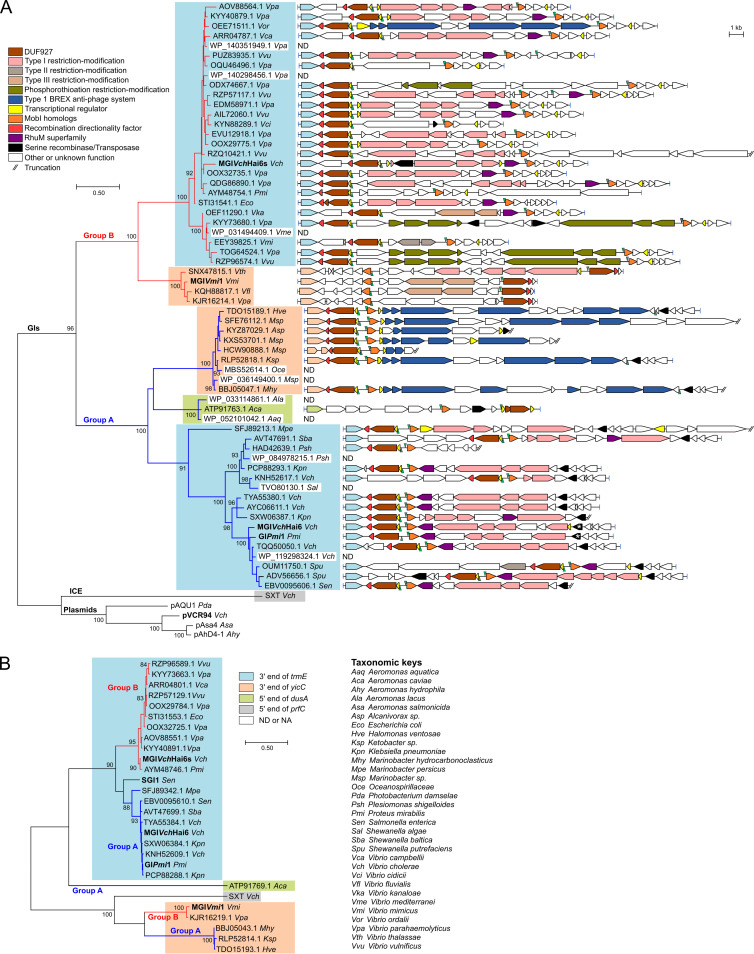
Diversity of genomic islands encoding MobI homologs. (A and B) Maximum likelihood phylogenetic analysis of MobI (A) and Int (B) homologs. Trees with the highest likelihoods (−7,967.92 and −5,688.94 for MobI and Int, respectively) are shown. Bootstrap supports are indicated as percentages at the branching points only when >80%. Branch lengths represent the number of substitutions per site over 201 and 311 amino acid positions for MobI and Int, respectively. Only one representative per cluster of similar proteins (>95% identity threshold) is shown in each tree. In panel A, the schematic structure of the genomic island encoding the corresponding MobI protein is shown next to each node. ORFs with similar function are color coded as indicated in the panel. Each node and the integrase gene of the corresponding node are color coded based on the integration site of the genomic island (refer to panel B for color key [ND, not determined; NA, not available]). Vertical green arrowheads indicate position and orientation of AcaCD binding sites. *attL* and *attR* attachment sites flanking each GI are represented by blue bars. The asterisks in MGI*Vch*Hai6 and GI*Pmi*1 indicate the insertion site of the complex resistance integrons. Additional details on GIs and host strains are provided in Table S1 in the supplemental material.

Given the importance of MobI-like factors, we searched the GenBank database for MobI homologs and extracted associated sequences to assess diversity of the GIs related to MGI*Vch*Hai6 (see Table S1 in the supplemental material). Phylogenetic analyses of MobI proteins revealed that MobI homologs encoded by GIs are distinct from those encoded by conjugative plasmids and cluster in two major groups (Fig. 5A). Group A contains GIs integrated at the 3′ end of *trmE* or *yicC*, and at the 5′ end of *dusA*. With the exception of the GI inserted at *dusA*, all GIs encoding group A MobI proteins exhibited the same structure: *int*-*xis*-*86*-*85*-*ig*-*mobI*, where *ig* likely contains the *oriT*, and *xis*-*86*-*85* and *mobI* are divergent and preceded by AcaCD-like binding sites. In a few GIs, variable DNA is inserted between the convergent *int* and *xis* genes. MGI*Vch*Hai6 and GI*Pmi*1 belong to group A. GI*Pmi*1 of Proteus mirabilis resembles MGI*Vch*Hai6, and it contains a large multidrug resistance cluster inserted at the same position but lacks the mercury resistance transposon Tn*6310* (19). Most *trmE*-specific group A GIs encode predicted type I restriction-modification (R/M) systems. In contrast, the *yicC*-specific group A GIs encode predicted type 1 BREX antiphage systems.

10.1128/mSphere.00748-20.4 TABLE S1 Features of GIs presented in Fig. 5 and accession numbers. Download Table S1, XLSX file, 0.03 MB. Copyright © 2020 Rivard et al. 2020 Rivard et al. This content is distributed under the terms of the Creative Commons Attribution 4.0 International license.

GIs coding for group B MobI proteins are integrated either at the 3′ end of *trmE* or 3′ end of *yicC*. In group B GIs, the *xis*-*86*-*85* cluster is separated from *mobI* by a large region of variable DNA often encoding predicted type I or phosphorothioation R/M systems. Furthermore, while *trmE*-specific group B GIs conserved the overall structure of group A GIs, *yicC*-specific group B GIs lack *85* and have undergone an inversion of the *xis*-*86*-to-*mobI* region relative to the *int* gene. These GIs encode predicted type I and type II R/M systems. Surprisingly, analysis undertaken in this study led to discovery of MGI*Vch*Hai6s, a group B GI inserted at *trmE* in a tandem fashion directly downstream of MGI*Vch*Hai6 in V. cholerae HC-36A1. Unlike MGI*Vch*Hai6, MGI*Vch*Hai6s does not carry antibiotic or heavy metal resistance genes. The group B GI of P. mirabilis JN40 encoding AYM48754.1 is also integrated in a tandem fashion downstream of SGI1-*Pm*JN40, an SGI1-like element that confers multidrug resistance (20). Thus, tandem integration of different GIs in *trmE* is not a rare occurrence.

Phylogenetic analysis of integrases encoded by respective GIs revealed similar clustering into groups A and B within *trmE*- and *yicC*-specific integrase clusters (Fig. 5B). Although the integrase encoded by SGI1 also mediates integration at *trmE*, it could not be ascribed to either group with confidence (<80 bootstraps), which is consistent with the considerable structural and genetic differences between SGI1-like and MGI*Vch*Hai6-like GIs.

## DISCUSSION

Bacterial conjugation typically results from concerted action of the cytoplasmic relaxosome responsible for DNA processing initiated at *oriT*, and the type IV secretion system (T4SS) that translocates the processed DNA across cell membranes into the recipient cell (21, 22). The type IV coupling protein (T4CP), an inner membrane-anchored protein, acts as a relaxosome docking station at the T4SS. In most cases, the relaxosome comprises a relaxase working together with auxiliary proteins that either help or are required for the relaxase activity. Most of the auxiliary factors hitherto described alter DNA topology by either locally bending DNA at the *oriT* or unwinding DNA through a helicase activity (23–29). Several *oriT*-binding factors, such as TraJ of RP4, TrwA of R388, and Int of Tn*916*, take part in specific recruitment of the relaxase at this locus (30–34). Other known auxiliary factors are involved in recognition of the T4CP or in relaxosome stabilization by protein-protein interactions (34–36).

Together with SXT/R391 elements, IncC and IncA plasmids share a set of transfer genes encoding a T4SS of the MPF_F_ family, a coupling protein (TraD), a relaxase of the MOB_H1_ family (TraI), and two essential factors thought to be part of the relaxosome (TraJ [DUF4400, PF14348] and MobI) (8, 10, 11, 37). We have also shown here that TraJ is essential to transfer of IncC plasmids and MGI*Vch*Hai6 (Fig. 4A and B). While results obtained with SXT and the IncHI1 plasmid R27 suggest a role as a relaxosome component (37–39), the exact function of TraJ remains unknown. MobI is required for conjugative transfer of IncC plasmids and SXT/R391 integrative and conjugative elements (ICEs) as well as GIs that mimic the *oriT* locus of the latter but lack a *mobI* gene (10, 40, 41). In contrast, deletion of *mobI* in the helper element does not abolish transfer of pCloDF13 and SGI1, which possess unique *oriT* loci and cognate mobilization proteins MobBC for pCloDF13 and MpsAB for SGI1 (12, 16, 40, 42). MGI*Vch*Hai6 lacks the mobilization genes *mpsAB* and the *oriT* locus that are essential for SGI1 mobilization (13, 16). Instead, we showed that MGI*Vch*Hai6 encodes MobI_M_, a distant homolog of the IncC plasmid-encoded MobI_C_ that provides some independence from *mobI_C_* (Fig. 1A, 2A, and 4B). Deletion of *mobI_C_* in the IncC plasmid R16a was shown to enhance (45-fold increase) mobilization of SGI1-C, suggesting competition between SGI1-C and its helper plasmid for the conjugative machinery (12). In contrast, we found that the absence of *mobI*_C_ impaired transfer of MGI*Vch*Hai6 (∼100-fold decrease) (Fig. 4B), suggesting that MobI_C_ enhances initiation of transfer mediated by TraI and MobI_M_ at *oriT* of MGI*Vch*Hai6. However, deletion of *mobI_M_*, while abolishing MGI*Vch*Hai6 mobilization, also enhanced transfer of the helper plasmid (12-fold increase) (Fig. 2A), consistent with competition between the two elements.

We identified the *oriT* locus of MGI*Vch*Hai6 within the intergenic region upstream of *mobI_M_* (*IG0*) and found that a 49-bp region (*IG8*) was sufficient to promote mobilization of a nonmobilizable plasmid. While *IG0* shares low nucleotide identity (45%) with the *oriT* of pVCR94 (10, 12), the 49-bp *IG8* shares 63% identity with this *oriT*, with the last 32 bp of *IG8* sharing 84% with the corresponding region in pVCR94 *oriT*. Nevertheless, this level of conservation is notably limited compared to the similarity reported between *oriT* of SXT and the GIs it mobilizes (>63% over ∼300 bp) (41). This lack of conservation likely accounts for the requirement for MobI_M_ to achieve optimal transfer of MGI*Vch*Hai6. Together with the absence of a MobI homolog in SXT-mobilizable GIs, the specificity of MobI_M_ and MobI_C_ for their respective elements further supports the proposed role for MobI as an auxiliary relaxosome component involved in *oriT* recognition (40, 43).

SGI1 encodes three functional T4SS subunits, TraH_S_, TraG_S_, and TraN_S_, that displace the homologous subunits encoded by IncC plasmids despite strong amino acid sequence divergences (64, 37, and 78% identity, respectively) (17). This alteration of the mating channel is crucial to enhance SGI1 mobilization (13, 17). Incorporation of TraG_S_ into the IncC mating pore allows SGI1 to circumvent entry exclusion exerted by an IncC plasmid in the recipient cells, allowing SGI1 to spread freely, even in a population of cells carrying IncC plasmids (17, 44). In contrast, MGI*Vch*Hai6 lacks genes encoding T4SS subunits. Predictably, MGI*Vch*Hai6 was shown here to conform to IncC entry exclusion and thus is unable to transfer to a strain already containing an IncC plasmid (see Fig. S2 in the supplemental material).

In SGI1, AcaCD binding sites have been identified upstream of *xis*, *S004*-*rep*, *traN_S_*, *traHG_S_*, and *S018* and corresponding promoters shown to respond to AcaCD activation (9, 17, 45–47). In MGI*Vch*Hai6, putative AcaCD binding sites were predicted at the 5′ end of *84* and upstream of an operon-like gene cluster containing *xis*, which encodes a putative protein sharing only 37% identity with the RDF Xis of SGI1 (13). In this study, Xis is indeed an RDF acting together with Int to catalyze excision under the control of AcaCD (Fig. 1B and C and Fig. S1). Regulation of *xis* and *mobI* by AcaCD is consistent with the previously proposed model in which these GIs remain quiescent in the chromosome in the absence of an IncC plasmid (43).

MGI*Vch*Hai6-like elements have been detected *in silico* in environmental and clinical O1 and non-O1/non-O139 V. cholerae isolates from the Indian subcontinent and South America, and in *Shewanella* sp. from North America, although those seem to be devoid of antibiotic resistance genes (13). IDH‐03944, a cotrimoxazole resistance-conferring GI related to MGI*Vch*Hai6, was recently reported in a 2011 isolate of V. cholerae O44 recovered from diarrheal patients in Kolkata, India (48). Comparative genomics revealed a much more diverse set of GIs related to MGI*Vch*Hai6 in the genome of marine dwelling species and integrated at the 3′ end of *trmE* and *yicC* or at the 5′ end of *dusA* (Fig. 5). The gene cargo of these GIs is predominantly associated with DNA modification (methylation, phosphorothioation) and restriction systems, as well as antiphage systems such as BREX (Fig. 5A). Only MGI*Vch*Hai6 and GI*Pmi*1, together with IDH‐03944 (48), contained integrons carrying antibiotic resistance genes, suggesting that these elements are an ancient, large reservoir of antiphage systems, recently hijacked as vectors for drug resistance genes. Most GIs of the MGI*Vch*Hai6 and MGI*Vch*Hai6s clades share a gene encoding a predicted RhuM-like virulence factor, usually immediately upstream or downstream of *mobI_M_* (Fig. 5). A mutant of *rhuM* located in S. enterica SPI-3 pathogenicity island is deficient for epithelial cell invasion, neutrophil transmigration, and killing of its Caenorhabditis elegans host (49). While these results suggest that MGI*Vch*Hai6-like elements may be involved in virulence modulation, the molecular function of RhuM is not known and its potency in the *Vibrionaceae* has not been assessed. One striking feature of MGI*Vch*Hai6-like elements is the syntenic conservation of an operon-like region typically including a predicted AcaCD binding site followed by genes encoding a putative transcriptional regulator containing a helix-turn-helix (HTH) domain, a DUF927 domain-containing protein, and the *xis* gene (Fig. 5A). While the DUF927 gene is ubiquitous, GIs of the MGI*Vmi*1 clade lack the upstream transcriptional regulator gene and the AcaCD binding site is located directly upstream of the DUF927 gene. Such high conservation is even more surprising since *85* and *86* are dispensable for mobilization of MGI*Vch*Hai6 by its helper plasmid (Fig. 1B). Why such factors should be under the control of the IncC transfer activator AcaCD is puzzling. Interestingly, a similar region exists in GIs mobilized by ICEs of the SXT/R391 family (Fig. S3). In prototypical MGI*Vfl*Ind1, a binding site of the transfer activator SetCD lies upstream of *rdfM* and *cds8*, which encode a predicted transcriptional regulator and a DUF927 domain-encoding protein. While the role of *cds8* remains elusive, RdfM shares weak amino acid identity (25%) with the product of *85* and acts as an RDF, facilitating excision of MGI*Vfl*Ind1 upon *setCD* expression (50). In addition to the aforementioned factors, MGI*Vfl*Ind1 and several MGI*Vch*Hai6-like GIs share an integration site at the 3′ end of *yicC* (Fig. 5B), an observation that prompted us to hypothesize that Xis and 85/RdfM act as alternative RDFs, each allowing excision from a specific integration site. To test this, a Δ*rdfM* mutant of MGI*Vfl*Ind1 was complemented by overexpressing *85* in a strain also containing ICE*Vfl*Ind1 to provide *setCD*. However, 85 failed to restore excision and an *attP* or *attB* junction was not detected (data not shown), suggesting that 85 has a different function or is too divergent to promote excision of MGI*Vfl*Ind1.

10.1128/mSphere.00748-20.3 FIG S3 Comparison of MGI*Vch*Hai6 and the conserved core of MGI*Vfl*Ind1. The maps are drawn to scale. The left and right junctions (*attL* and *attR*) within the host chromosome are indicated by blue ticks at the extremities. ORFs with similar function are color coded as indicated in the legend. Green arrows indicate the position and orientation of AcaCD and SetCD binding sites. Homologous regions are bracketed and linked by a gray line with the corresponding percentage of nucleotide identity. MGI*Vfl*Ind1 can be accessed through GenBank accession number KC117176. Download FIG S3, PDF file, 0.07 MB. Copyright © 2020 Rivard et al. 2020 Rivard et al. This content is distributed under the terms of the Creative Commons Attribution 4.0 International license.

SXT/R391 ICEs have been shown to promote conjugative transfer of chromosomal DNA located 3′ of *prfC*, their integration site, and remotely 5′ of *yicC*, the integration site of the GIs they mobilize (41, 51). Thus, these elements are able to mobilize large stretches (≥1Mbp) of chromosomal DNA locally and remotely in an Hfr-like manner, suggesting that SXT/R391 ICEs play an evolutionary role that extends beyond their own dissemination. Our results show that IncC plasmids can mobilize chromosomal DNA located downstream of *trmE* by mobilization of MGIs without their prior excision from the chromosome (Fig. 3). Given the presence of MGI*Vch*Hai6-like elements integrated at different chromosomal sites and across a wide range of *Vibrionaceae* and other species of *Gammaproteobacteria*, IncC plasmids and their subordinate GIs can be concluded to comprise a potent driving force in the gene flow circulating in many bacterial pathogens. In fact, this is superbly exemplified by the recently reported presence of an MGI*Vch*Hai6-like GI in V. cholerae Santiago-089, a non-O1/non-O139 clinical isolate harboring many virulence genes scattered throughout chromosome 1 (52). Not only is the GI itself poised to be mobilized by an incoming IncC plasmid—along with the GI-borne antibiotic and mercury resistance genes—but it is also plausible that it may, in fact, usher transfer of downstream elements GI*Vch*-T3SS and VPI-2, thus simultaneously contributing to dissemination of virulence determinants.

Cholera continues to cause epidemics that include millions of cases worldwide (53). The geographical range of V. cholerae is expected to expand dramatically as climate change renders the marine environment increasingly hospitable to this pathogen (54, 55). While the ability to promote epidemic outbreaks was traditionally regarded as an appanage of O1 and O139 strains (56), it is becoming increasingly clear that the actual picture is far more nuanced (52, 57–60). In various species, cumulative acquisition of antibiotic resistance and/or virulence determinants through exchange of genomic islands has time and again allowed emergence of virulent strains, some of which lack canonical virulence hallmarks (61–63). IncC plasmids circulating in non-O1/non-O139 V. cholerae populations may prove to comprise the perfect trigger for emergence of unforeseen pandemics.

## MATERIALS AND METHODS

### Bacterial strains and media.

The bacterial strains and plasmids used in this study are described in Table 1. The strains were routinely grown in lysogeny broth (LB) (EMD) at 37°C in an orbital shaker and stored at −80°C in LB broth with 15% (vol/vol) glycerol. The following antibiotics and concentrations were employed: ampicillin (Ap), 50 μg/ml; chloramphenicol (Cm), 20 μg/ml; kanamycin (Kn), 10 μg/ml for single-copy integrants of pOP*lacZ*-derived constructs, 50 μg/ml otherwise; nalidixic acid (Nx), 40 μg/ml; rifampin (Rf), 50 μg/ml: spectinomycin (Sp), 50 μg/ml; streptomycin (Sm), 200 μg/ml; tetracycline (Tc), 12 μg/ml. Conjugation assays were performed as previously described (17). However, donors and recipients were selected according to their sole chromosomal markers. When required, mating experiments were performed using LB plates with 0.02% arabinose to induce expression of pBAD30-derived complementation vectors. The frequencies of transconjugant formation were computed as ratios of transconjugant per donor CFU from three independent mating experiments.

**TABLE 1:** Strains and plasmids used in this study

Strain, plasmid, or element	Relevant genotype or phenotype^*a*^	Reference
Vibrio cholerae		
HC-36A1	Clinical, non-O1/non-O139, Haiti, 2010 (Ap Cm Kn Sp Sm Su Tc Tm)	57
Escherichia coli		
BW25113	F^−^ Δ(*araD*-*araB*)*567* Δ*lacZ4787*(::*rrnB-3*) λ^−^ *rph-1* Δ(*rhaD*-*rhaB*)*568 hsdR514*	75
GG56	Nx^r^ derivative of BW25113 (Nx)	76
VB112	Rf^r^ derivative of MG1655 (Rf)	40
CAG18439	MG1655 *lacZU118 lacI42*::Tn*10* (Tc)	77
VB47	CAG18439 Δ*recA* Δ*galK*	78
JW3642	BW25113 Δ*uhpC772*::*aph* (Kn)	18
JW3692	BW25113 Δ*purP745*::*aph* (Kn)	18
JW3718	BW25113 Δ*gidB769*::*aph* (Kn)	18
JW5858	BW25113 Δ*yidX732*::*aph* (Kn)	18
Plasmids		
pVCR94	IncC conjugative plasmid, V. cholerae O1 El Tor (Su Tm Cm Ap Tc Sm)	10
pVCR94^Sp^	Sp^r^ derivative of pVCR94 (pVCR94ΔX2) (Su Sp)	9
pVCR94^Kn^	Kn^r^ derivative of pVCR94 (pVCR94ΔX3) (Su Kn)	9
pVCR94^Cm^	Cm^r^ derivative of pVCR94 (pVCR94ΔX4) (Su Cm)	17
pVCR94Δ*mobI_C_*	*mobI_C_* deletion mutant of pVCR94^Sp^ (Su Sp)	This study
pVCR94Δ*traI*	*traI* deletion mutant of pVCR94^Sp^ (Su Sp)	This study
pVCR94Δ*traD*	*traD* deletion mutant of pVCR94^Sp^ (Su Sp)	This study
pVCR94Δ*traJ*	*traJ* deletion mutant of pVCR94^Sp^ (Su Sp)	This study
pVCR94Δ*traN*	*traN* deletion mutant of pVCR94^Sp^ (Su Sp)	9
pVCR94Δ*traG*	*traG* deletion mutant of pVCR94^Sp^ (Su Sp)	9
pVCR94Δ*traH*	*traH* deletion mutant of pVCR94^Sp^ (Su Sp)	9
pBAD30	*ori*_p15A_ *bla araC P*_*BAD*_ (Ap)	79
p*acaCD*	pBAD30::*acaDC* (Ap)	9
pBAD-*mobI_C_*	pBAD30::*mobI_C_* (Ap)	This study
pBAD-*traD*	pBAD30::*traD* (Ap)	This study
pBAD-*traJ*	pBAD30::*traJ* (Ap)	This study
pBAD-*traN*	pBAD30::*traN* (Ap)	17
pBAD-*traG*	pBAD30::*traG* (Ap)	17
pBAD-*traH*	pBAD30::*traH* (Ap)	17
pBAD-*int*	pBAD30::*int* (Ap)	This study
pBAD-*xis*	pBAD30::*xis* (Ap)	This study
pT84	pBAD-TOPO::*84* (Ap)	This study
pBAD-*84*	pBAD30::*84* (Ap)	This study
pBAD-*84*-31	pBAD30::*84* starting at position +31 of *84* (Ap)	This study
pBAD-*84*-106	pBAD30::*84* starting at position +106 of *84* (Ap)	This study
pBAD-*84*-118	pBAD30::*84* starting at position +118 of *84* (Ap)	This study
pBAD-*84*-304	pBAD30::*84* starting at position +304 of *84* (Ap)	This study
pBAD-*84*-409	pBAD30::*84* starting at position +409 of *84* (Ap)	This study
pOP*lacZ*	*ori*_R6Kγ_ *attP*_λ_ *aph lacZ* (Kn)	9
pProm*int*	pOP*lacZ* containing −163 to −12 of MGI*Vch*Hai6 *P_int_* (Kn)	This study
pProm*85*	pOP*lacZ* containing −389 to −11 of MGI*Vch*Hai6 *P_85_* (Kn)	This study
pProm*mobI*	pOP*lacZ* containing −345 to −11 of MGI*Vch*Hai6 *P_84_* (Kn)	This study
pProm*traH*_*S*_	pOP*lacZ* containing −243 to −14 of SGI1 *P_traHs_* (Kn)	17
pDC1	pACYC184 Δ*cat* (ΔMscI-PvuII) (Tc)	40
p*IG0*-*84*	pDC1::*IG0*-*84* (Tc)	This study
p*IG0*-*84*G120C	pDC1::*IG0*-*84* G120C (Tc)	This study
p*IG0*-*84*G306C	pDC1::*IG0*-*84* G306C (Tc)	This study
p*IG0*	pDC1::*IG0* (Tc)	This study
p*IG1*	pDC1::*IG1* (Tc)	This study
p*IG2*	pDC1::*IG2* (Tc)	This study
p*IG3*	pDC1::*IG3* (Tc)	This study
p*IG4*	pDC1::*IG4* (Tc)	This study
p*IG5*	pDC1::*IG5* (Tc)	This study
p*IG6*	pDC1::*IG6* (Tc)	This study
p*IG7*	pDC1::*IG7* (Tc)	This study
p*IG8*	pDC1::*IG8* (Tc)	This study
p*IG9*	pDC1::*IG9* (Tc)	This study
p*IG10*	pDC1::*IG10* (Tc)	This study
p*IG11*	pDC1::*IG11* (Tc)	This study
p*IG12*	pDC1::*IG12* (Tc)	This study
p*IG13*	pDC1::*IG13* (Tc)	This study
pINT-ts	*oriR101* *cI857* λ*p*_R_-*int*_λ_ (ts, Ap)	80
pSIM6	λRed recombination thermo-inducible encoding plasmid (ts, Ap)	81
pMS1	λRed recombination thermo-inducible encoding plasmid (ts, Gm)	10
pKD3	*cat* (Cm) template for one-step chromosomal gene inactivation	75
pKD4	*aph* (Kn) template for one-step chromosomal gene inactivation	75
pCP20	Flp recombinase thermo-inducible encoding plasmid (ts, Ap Cm)	82
pCP20-Gm	Gm^r^ derivative of pCP20 (ts, Gm Cm)	83
Genomic islands		
MGI*Vch*Hai6	Drug resistance island, V. cholerae HC-36A1 (Ap Cm Sm Su Tm Tc)	13
MGI^Kn^	Kn^r^ derivative of MGI*Vch*Hai6 lacking In36A1 and Tn*6310* (Kn)	This study
MGI^Cm^	Cm^r^ derivative of MGI*Vch*Hai6 lacking In36A1 and Tn*6310* (Cm)	This study
MGI^Kn^Δ*int*	*int* deletion mutant of MGI^Kn^ (Kn)	This study
MGI^Kn^Δ*xis*	*xis* deletion mutant of MGI^Kn^ (Kn)	This study
MGI^Cm^Δ*xis*	*xis* deletion mutant of MGI^Cm^ (Cm)	This study
MGI^Kn^Δ*86*	*86* deletion mutant of MGI^Kn^ (Kn)	This study
MGI^Kn^Δ*85*	*85* deletion mutant of MGI^Kn^ (Kn)	This study
MGI^Kn^Δ*84*	*84* deletion mutant of MGI^Kn^ (Kn)	This study

a Ap, ampicillin; Cm, chloramphenicol; Gm, gentamicin, Kn, kanamycin; Nx, nalidixic acid; Rf, rifampin; Sp, spectinomycin; Sm, streptomycin; Su, sulfamethoxazole; Tc, tetracycline; Tm, trimethoprim; ts, thermosensitive.

### Molecular biology methods.

Plasmid DNA was extracted using the EZ-10 Spin Column Plasmid DNA Minipreps kit (Bio Basic) following the manufacturer’s instructions. Enzymes used in this study were purchased from New England Biolabs. PCR assays were performed with the primers listed in Table 2. PCR conditions were as follows: (i) 3 min at 94°C; (ii) 30 cycles, with 1 cycle consisting of 30 s at 94°C, 30 s at the appropriate annealing temperature, and 1 min/kb at 68°C; and (iii) 5 min at 68°C. When required, the resulting products were purified using the EZ-10 Spin Column PCR Products purification kit (Bio Basic) following the manufacturer’s instructions. E. coli strains were transformed by electroporation as described previously (64) in a Bio-Rad Gene Pulser Xcell device set at 25 μF, 200 V, and 1.8 kV using 1-mm gap electroporation cuvettes.

**TABLE 2:** DNA sequences of the primers used in this study

Primer name	Nucleotide sequence (5′ to 3′)^*a*^	Reference
94delmobI.for	ATGTGGATAGGAATTGGATAGGAATTGGGAGGGTATTGAGGTGTAGGCTGGAGCTGCTTC	This study
94delmobI.rev	TCCCCAGTTTCGCCAATTCAGTGGCCGCTACAGATGCTGTCATATGAATATCCTCCTTA	This study
94deltraI.for	TGTAGCGACGAGAAATACGATAGGAATATGGTCAACCTACGTGTAGGCTGGAGCTGCTTC	This study
94deltraI.rev	CACGGCATCTCGTAGGCGAGCGGGTCATAACTCATTGTCATATGAATATCCTCCTTA	This study
94deltraD.for	CAGGGAAAATTGTATTTAGAGGTAAATGAAACATGAGTGTAGGCTGGAGCTGCTTC	This study
94deltraD.rev	TCTCTATACTTCTTGTCACACATAGCTCTGCTCTTACATATGAATATCCTCCTTA	This study
94deltraJ.for	GCCGTGGTTGCTGGTCGCATGGCTGCTGGTTATTGAGTGTAGGCTGGAGCTGCTTC	This study
94deltraJ.rev	ATTTATAACGGAGTAGCTCATCCTCCCTCCCTATACCATATGAATATCCTCCTTA	This study
Hai6delWE.for	TGCCGTAATCAAGGAAATGATACGGCAGAAAGTCGTGGAACGGAATAGGAACTTCAAGAT	This study
Hai6delWE.rev	TGATTACGACTACCGAAACCATATTGGGTTTAATGACTGAAGGAACTTCAGAGCGCTT	This study
Hai6delint.for	CTGGCGCACATATGGCGCACAAGATGAGAAAAACCTGTGTAGGCTGGAGCTGCTTC	This study
Hai6delint.rev	TTGTAAAGATGTTGTCGTTTGATCGTAATAATGCTCTTACATATGAATATCCTCCTTA	This study
Hai6delxis.for	TCCCACTTTAAGTTGGCAGTGTAGGAGAGTATCAACGTGTAGGCTGGAGCTGCTTC	This study
Hai6delxis.rev	CCATTCATATTTATTTTCAAAAGGTTAAATCTCAACTTACATATGAATATCCTCCTTA	This study
Hai6del86.for	GCTGCATGGTTGAGTGATTTATAAAAGGGGGAAGTGGTGTAGGCTGGAGCTGCTTC	This study
Hai6del86.rev	TTAAAGTGGGATAAACGGGAAACTGGGAACACTAAGTTACATATGAATATCCTCCTTA	This study
Hai6del85.for	TTACTCCTGCAATCTAGATAATAAACAGGAGCCATCGTGTAGGCTGGAGCTGCTTC	This study
Hai6del85.rev	CTGCAACTTTTTTTGCATCACTTCCCCCTTTTATAACATATGAATATCCTCCTTA	This study
Hai6delmobI.for	ATCAGATAGTTATTGGAAAGGAATTGGTAGGGTCTTGTGTAGGCTGGAGCTGCTTC	This study
Hai6delmobI.rev	CTCTAATGAGTTGAGGGCTGTGTTGTAAGATCATAATTACATATGAATATCCTCCTTA	This study
94mobIEcoRI.for	NNNNNN GAATTC AAGGAGGAATAATAAATGAGTCTACCAACAGAGCGATGGCT	This study
94mobIEcoRI.rev	NNNNNN GAATTC TCACACCTCGTCGCTATGTGTCTT	This study
94traD61EcoRI.for	NNNN GAATTC AAGGAGGAATAATAAATGACAATGAGTTATGAC	This study
94traD61EcoRI.rev	NNNN GAATTC TTACTGGGTCAATATGGGCAGAC	This study
94traJ63EcoRI.for	NNNN GAATTC AAGGAGGAATAATAAATGAAGAAGCCGTGGTTGCTG	This study
94traJ63EcoRI.rev	NNNN GAATTC CTATACCCGTTTCTGGAGGTTG	This study
Hai6intEcoRI.for	NNNN GAATTC AAGGAGGAATAATAAATGAAAATCAGCATACATAAAC	This study
Hai6intEcoRI.rev	NNNN GAATTC TTAGTCTGATGACTCATCGAAG	This study
Hai6xisEcoRI.for	NNNN GAATTC AAGGAGGAATAATAAATGACTGAAAAAGAGATGCTTC	This study
Hai6xisEcoRI.rev	NNNN GAATTC CTAATATCTCCGGCCATAC	This study
Hai6mobIXbaI.for	NNNNNN TCTAGA AAGGAGGAATAATAAATGGCTAAGGCGTCACAACAAAA	This study
Hai6mobIXbaI.rev	NNNNNN TCTAGA TTATAAAGGATTATCCATCTTAAAATCGTCGAT	This study
Hai6mobINcoI.for	CCATGG CTAAGGCGTCACAAC	This study
Hai6mobI.rev	TTATAAAGGATTATCCATCTTAAAATCGTCGAT	This study
Hai6promintPstI.f	NNNNNN CTGCAG AATAGTAAAACACATCAAACC	This study
Hai6promintPstI.r	NNNNNN CTGCAG ATCTTGTGCGCCATATGTGCG	This study
Hai6prom85PstI.f	NNNNNN CTGCAG AATAACTATCTGATGGGGGAT	This study
Hai6prom85PstI.r	NNNNNN CTGCAG GTTTATTATCTAGATTGCAGG	This study
Hai6prom84PstI.f	NNNNNN CTGCAG AATGAGTTGCTCGACATTAC	This study
Hai6prom84PstI.r	NNNNNN CTGCAG ACTGTATGTTGAATACAGAG	This study
43_attPF	GCAATTAATGATAAAGACGGGTA	13
Ec104D.rev	AACCATTTTGAGGTCACACA	15
Hai6_attPR	TCAAATCACTTCCCACCAAG	This study
Start_mobI_F1	GTGCCCCAAAAGGGCACGAAG	This study
Start_mobI_F2	GTGGATTATGTCATGACCAA	This study
Start_mobI_F3	ATGACCAAAAATTATTCATTTTTAG	This study
Start_mobI_F4	ATGCTTTTTTCATTATCAGTTTCAG	This study
Start_mobI_F5	TTGTTCTGGCAGCACAACCGA	This study
Start_mobI_Rv2	TTATTATTCCTCCTTTCTAGAGGATC	This study
Hai6oriT84XbaI.for	NNNNNN TCTAGA TTGCAGGAGTAATCATCTCGAAAAA	This study
mobI120_GC.F	ATTATGTCATCACCAAAAATTATTCATTTTTAG	This study
mobI120_GC.R	CCACTGTATGTTGAATACAG	This study
mobI306_GC.F	ACGATGCTATCCTTTTTTCATTATC	This study
mobI306_GC.R	CTTGATTAACGTTTGTGC	This study
Hai6oriTXbaI.rev	NNNNNN TCTAGA AAGACCCTACCAATTCCTTTC	This study
minoriT1.F	CACGCTGATATTTAATGATATTTATTC	This study
minoriT1-4a.R	TCTAGAAATATTTTATCTGATTAATAAGATG	This study
minoriT2.F	AGCCACCAATGAGTTGCT	This study
minoriT3.F	CCGTATCAATTTATGCGC	This study
minoriT4b.F	TCTAGATTTCAGTGCAATTTATC	This study
minoriT4b.R	ACGATTTATTCCATACAAGC	This study
minoriT5.F	CTCAGGTCTTTTATTGGC	This study
minoriT5.R	CTCTGGTTATTCACTGGC	This study
minoriT7.F	AATAAATCGTCCAAGATCC	This study
minoriT8.F	TCCAAGATCCCCCATCAG	This study
minoriT8XbaI.F	NNNN TCTAGA TCCAAGATCCCCCATCAG	This study
minoriT9XbaI.R	NNNN TCTAGA GACATAATCCACTGTATGTTG	This study
minoriT11.R	ACGGGTCTTTTGTTGTGAC	This study
Hai6delWEcm.for	TGCCGTAATCAAGGAAATGATACGGCAGAAAGTCGTGGAATACCTGTGACGGAAGATCAC	This study
Hai6delWEcm.rev	TGATTACGACTACCGAAACCATATTGGGTTTAATGACTGATAGGAACTTCATTTAAATGG	This study

a Restriction sites are underlined.

### Plasmid and strain construction.

The plasmids and primers used in this study are listed in Tables 1 and 2, respectively. Detailed description of plasmid and strain construction is provided in Text S1 in the supplemental material.

10.1128/mSphere.00748-20.5 TEXT S1 Plasmid and strain construction. Download Text S1, DOCX file, 0.02 MB. Copyright © 2020 Rivard et al. 2020 Rivard et al. This content is distributed under the terms of the Creative Commons Attribution 4.0 International license.

### Detection of MGI*Vch*Hai6 excision.

Excision of MGI*Vch*Hai6 was detected by PCR on genomic DNA of the appropriate strains using primers listed in Table 2. The *attR* and *attP* sites were respectively amplified using primer pairs 43_attPF/Ec104D.rev and 43_attPF/Hai6_attPR.

### β-Galactosidase assays.

Qualitative assays were performed by depositing 10-μl aliquots of overnight cultures with appropriate antibiotics on solid agar supplemented with 5-bromo-4-chloro-3-indolyl-β-d-galactopyranoside (X-gal) with or without 0.02% arabinose. The plates were observed after an overnight incubation at 37°C.

Quantitative assays were performed with 2-nitrophenyl-β-d-galactopyranoside (ONPG) according to a protocol adapted from Miller (65). After an overnight incubation at 37°C with appropriate antibiotics, cultures were diluted 1:100 in 50 ml LB broth supplemented with 50 μg/ml ampicillin and grown until an optical density at 600 nm (OD_600_) of 0.2 was reached. Two series of 1/10 dilutions were then prepared in total volumes of 5 ml LB broth supplemented with 50 μg/ml ampicillin with or without 0.2% arabinose and incubated for 2 h at 37°C.

### Phylogenetic analyses.

The primary sequence of homologs of MobI proteins encoded by MGI*Vch*Hai6 and MGI*Vmi*1 were obtained using the NCBI blastp algorithm (66) against the nr/nt database restricted to *Gammaproteobacteria* (taxid: 1236). Primary sequences sharing less than 45% identity and under 85% minimum coverage were filtered out of subsequent analyses. Distant MobI homologs from SXT (GenBank accession no. EET25017.1), pVCR94 (GenBank WP_001447712.1), pAhD4-1 (GenBank ALZ82609.1), pAsa4 (GenBank ABO92354.1), and pAQU1 (GenBank WP_014386842.1) were introduced manually in the data set as an outgroup. MobI homologs were first clustered with CD-HIT (67) to the best cluster that met the 0.95 identity cutoff prior to alignment. The predicted primary sequences of Int homologs were recovered from a representative sample of GIs encoding MobI homologs. Int primary sequences of SXT and SGI1 (GenBank accession no. AF261825.2) were introduced manually. Phylogenetic analyses were computed using amino acid alignments generated by MUSCLE (68). Prior to phylogenetic analysis, poorly aligned regions were discarded using trimAl v1.3 software with the automated heuristic approach (69). Evolutionary analyses were performed within MEGA X (v 10.0.5) (70) using the maximum likelihood method (PhyML) (71) and either the JTT (MobI) or LG (Int) matrix-based models (72, 73). The initial tree(s) for the heuristic search was obtained automatically by applying neighbor-joining and BioNJ algorithms to a matrix of pairwise distances estimated using a JTT model and then selecting the topology with superior log likelihood value.
