# Supplementary figures and images for: Antibiotic Resistance in Vibrio cholerae: Mechanistic Insights from IncC Plasmid-Mediated Dissemination of a Novel Family of Genomic Islands Inserted at trmE
Source: mSphere. 2020 Aug 26;5(4):e00748-20. doi: 10.1128/mSphere.00748-20 (PMC7449626; doi:10.1128/mSphere.00748-20)

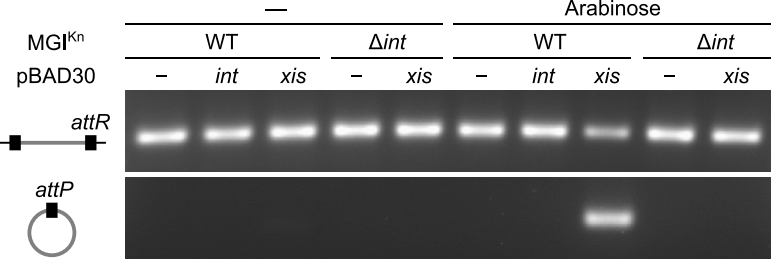

Supplement: FIG S1 [file mSphere.00748-20-sf001.pdf]

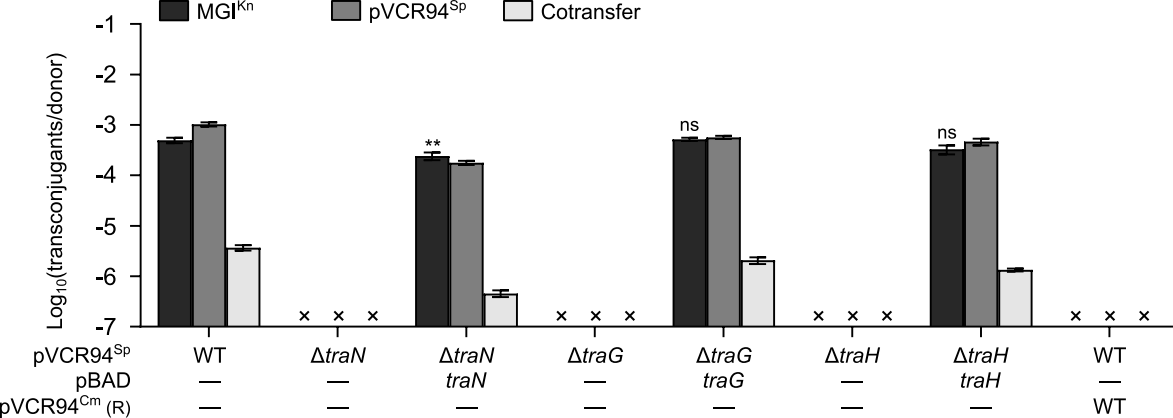

Supplement: FIG S2 [file mSphere.00748-20-sf002.pdf]
