# Supplementary material for: Antibiotic Resistance in Vibrio cholerae: Mechanistic Insights from IncC Plasmid-Mediated Dissemination of a Novel Family of Genomic Islands Inserted at trmE
Source: mSphere. 2020 Aug 26;5(4):e00748-20. doi: 10.1128/mSphere.00748-20 (PMC7449626; doi:10.1128/mSphere.00748-20)
Supplement: FIG S3 [file mSphere.00748-20-sf003.pdf]

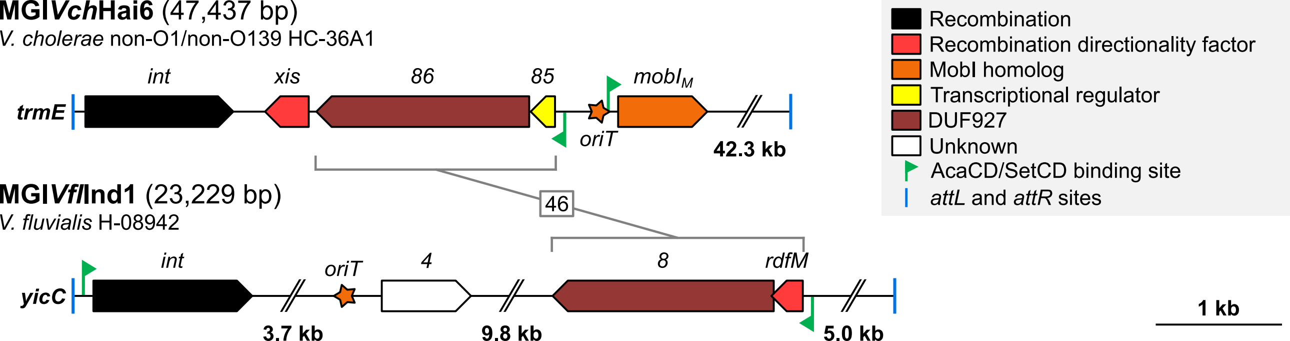
MGIVchHai6 (47,437 bp)
V. cholerae non-O1/non-O139 HC-36A1
int
xis
86
85
mobI$_M$
trmE
oriT
42.3 kb
46
MGIVflInd1 (23,229 bp)
V. fluvialis H-08942
int
oriT
4
8
rdfM
yicC
3.7 kb
9.8 kb
5.0 kb
1 kb
Recombination
Recombination directionality factor
MobI homolog
Transcriptional regulator
DUF927
Unknown
AcaCD/SetCD binding site
attL and attR sites
