# Supplementary material for: Antibiotic Resistance in Vibrio cholerae: Mechanistic Insights from IncC Plasmid-Mediated Dissemination of a Novel Family of Genomic Islands Inserted at trmE
Source: mSphere. 2020 Aug 26;5(4):e00748-20. doi: 10.1128/mSphere.00748-20 (PMC7449626; doi:10.1128/mSphere.00748-20)
Supplement: TEXT S1 [file mSphere.00748-20-s0001.docx]

**Supplemental Text S1**

Antibiotic Resistance in *Vibrio cholerae*: Mechanistic Insights from IncC Plasmid-Mediated Dissemination of a Novel Family of Genomic Islands Inserted at *trmE*

# Experimental Procedures

## **Plasmids and strains construction**

Plasmids used for complementation assays were derived from pBAD30. *mobI*_C_, *traD* and *traJ* were amplified using the primer pairs 94mobIEcoRI.for/ 94mobIEcoRI.rev, 94traD61EcoRI.for/94traD61EcoRI.rev and 94traJ63EcoRI.for/94traJ63EcoRI.rev, and genomic DNA from BW25113 Nx containing pVCR94ΔX2 as the template. The resulting amplicons were digested with EcoRI and cloned into EcoRI-digested pBAD30 using T4 DNA ligase, generating pBAD-*mobI*_C_, pBAD-*traD* and pBAD-*traJ*. Similarly, *int*, *xis* and *84* were amplified using the primer pairs Hai6intEcoRI.for/Hai6intEcoRI.rev, Hai6xisEcoRI.for/Hai6xisEcoRI.rev and Hai6mobIXbaI.for/Hai6mobIXbaI.rev, and genomic DNA from HC-36A1 as the template. The amplicons were digested with EcoRI or XbaI and cloned into EcoRI- or XbaI-digested pBAD30 using T4 DNA ligase, generating, pBAD-*int*, pBAD-*xis* and pBAD-*84*. To construct pT84, *84* was amplified with the primer pair Hai6mobINcoI.for/Hai6mobI.rev and cloned into pBAD-TOPO using the pBAD-TOPO TA Expression Kit (Thermo Fisher) following the manufacturer’s instructions, and the N-terminal leader sequence was removed by NcoI digestion.

pBAD-*84*-31, pBAD-*84*-106, pBAD-*84*-118, pBAD-*84*-304 and pBAD-*84*-409 were generated using the Q5 Site-Directed Mutagenesis Kit (New England Biolabs) following the manufacturer’s instruction, with pBAD-*84* as the template and the primer pairs Start_mobI_F1/Start_mobI_Rv2, Start_mobI_F2/Start_mobI_Rv2, Start_mobI_F3/Start_mobI_Rv2, Start_mobI_F4/Start_mobI_Rv2, Start_mobI_F5/Start_mobI_Rv2, respectively.

The *IG0*-*84* locus was amplified using the primer pair Hai6oriT84XbaI.for/Hai6mobIXbaI.rev and HC-36A1 as template. The resulting amplicon was digested with XbaI and cloned into XbaI-digested pDC1 using T4 DNA ligase, generating p*IG0*-*84*. p*IG0*-*84*G120C and p*IG0*-*84*G306C were generated using the Q5 Site-Directed Mutagenesis Kit (New England Biolabs) following the manufacturer’s instruction, with p*IG0*-*84* as the template and the primer pairs mobI120_GC.F/mobI120_GC.R and mobI306_GC.F/mobI306_GC.R, respectively.

*IG0*, *IG9* and *IG10* were amplified using the primer pairs Hai6oriT84XbaI.for/Hai6oriTXbaI.rev, minoriT8XbaI.F/minoriT9XbaI.R and Hai6oriT84XbaI.for/minoriT9XbaI.R. The resulting amplicons were digested with XbaI and cloned into XbaI-digested pDC1 using T4 DNA ligase, generating p*IG0*, p*IG9* and p*IG10*. p*IG1*, p*IG2*, p*IG3*, p*IG4*, p*IG5*, p*IG6*, p*IG7* and p*IG8* were generated using the Q5 Site-Directed Mutagenesis Kit (New England Biolabs) following the manufacturer’s instruction, with p*IG0* as the template and the primer pairs minoriT1.F/minoriT1-4a.R, minoriT2.F/minoriT1-4a.R, minoriT3.F/minoriT1-4a.R, minoriT4b.F/minoriT4b.R, minoriT5.F/minoriT5.R, minoriT5.F/minoriT1-4a.R, minoriT7.F/minoriT1-4a.R and minoriT8.F/minoriT1-4a.R, respectively. Similarly, p*IG11*, p*IG12* and p*IG13* were generated with p*IG10*, p*IG11* and p*IG9* as respective templates using primer pairs minoriT4b.F/minoriT11.R, minoriT5.F/minoriT1-4a.R and minoriT4b.F/minoriT11.R, respectively.

Fragments encompassing promoter regions upstream of *int*, *85* and *mobI*_M_ were amplified using primer pairs Hai6promintPstI.f/Hai6promintPstI.r, Hai6prom85PstI.f/Hai6prom85PstI.r and Hai6prom84PstI.f/Hai6prom84PstI.r, and genomic DNA from HC-36A1 as template. The amplicons were digested with PstI and cloned into PstI-digested pOP*lacZ* to produce pProm*int*, pProm*85* and pProm*mobI*, respectively (1). The resulting constructs were single-copy integrated into the *attB*_λ_ chromosomal site of BW25113 using pINT-ts (2).

All constructs were verified by PCR and DNA sequencing by Plateforme de Séquençage et de Génotypage du Centre de Recherche du CHUL (Québec, QC, Canada).

Deletion mutants of pVCR94^Sp^ and MGI*Vch*Hai6 (Table 1) were constructed using the one-step chromosomal gene inactivation method (3) and primers listed in Table 2. All deletions were designed to be non-polar. Deletion mutants of *mobI*_C_, *traI*, *traD* and *traJ* in pVCR94^Sp^ were obtained using primer pairs 94delmobI.for/ 94delmobI.rev, 94deltraI.for/ 94deltraI.rev, 94deltraD.for/ 94deltraD.rev and 94deltraJ.for/ 94deltraJ.rev, respectively, and pKD3 as the template. MGI^Kn^ and MGI^Cm^ were obtained using the primer pairs Hai6delWE.for/Hai6delWE.rev and Hai6delWEcm.for/Hai6delWEcm.rev, and pKD4 and pKD3 as the templates, respectively. Subsequent deletions of *int*, *xis*, *86*, *85* and *84* in MGI^Kn^ or MGI^Cm^ were obtained using primer pairs Hai6delint.for/Hai6delint.rev, Hai6delxis.for/Hai6delxis.rev, Hai6del86.for/Hai6del86.rev, Hai6del85.for/Hai6del85.rev and Hai6delmobI.for/Hai6delmobI.rev, respectively, and pKD3 as the template. The λRed recombination system was expressed using either pSIM6 or pMS1. When appropriate, resistance cassettes were excised from the resulting constructions using the Flp-encoding plasmids pCP20 or pCP20-Gm (4, 5). All deletions were validated by antibiotic profiling and PCR.

# References

1. Carraro N, Matteau D, Luo P, Rodrigue S, Burrus V. 2014. The master activator of IncA/C conjugative plasmids stimulates genomic islands and multidrug resistance dissemination. PLoS Genet 10:e1004714.

2. Haldimann A, Wanner BL. 2001. Conditional-replication, integration, excision, and retrieval plasmid-host systems for gene structure-function studies of bacteria. J Bacteriol 183:6384–6393.

3. Datsenko KA, Wanner BL. 2000. One-step inactivation of chromosomal genes in *Escherichia coli* K-12 using PCR products. Proc Natl Acad Sci U S A 97:6640–6645.

4. Cherepanov PP, Wackernagel W. 1995. Gene disruption in *Escherichia coli*: TcR and KmR cassettes with the option of Flp-catalyzed excision of the antibiotic-resistance determinant. Gene 158:9–14.

5. Doublet B, Douard G, Targant H, Meunier D, Madec J-Y, Cloeckaert A. 2008. Antibiotic marker modifications of lambda Red and FLP helper plasmids, pKD46 and pCP20, for inactivation of chromosomal genes using PCR products in multidrug-resistant strains. J Microbiol Methods 75:359–361.
